# The damage potential of near-wall shock waves from cavitation bubbles investigated via total internal reflection fluorescence imaging

**DOI:** 10.1016/j.ultsonch.2026.108044

**Published:** 2026-09-08

**Authors:** Fangyi Wang, Sebastian A. Kaiser

**Affiliations:** EMPI, Institute for Energy and Materials Processes—Reactive Fluids, University of Duisburg-Essen, 47057 Duisburg, Germany

**Keywords:** Laser-induced cavitation, Total internal reflection fluorescence, Evanescent wave microscopy, Shock-wave velocity, Shock-wave pressure

## Abstract

This study establishes total internal reflection fluorescence (TIRF) imaging as a diagnostic method for investigating shock waves generated by collapsing laser-induced cavitation bubbles near a solid–liquid interface. TIRF was combined with synchronized short-pulse transillumination to capture both near-wall shock signatures and conventional bubble dynamics. A temperature-compensating Rhodamine 6G/Rhodamine B mixture made fluorescence variations primarily sensitive to density- and refractive-index changes. By tuning the laser incidence angle relative to the critical angle, the evanescent-field depth and density sensitivity were controlled, enabling robust detection of shock-wave footprints at the wall. Bubbles of 1–1.5 mm radius at moderate stand-off distances γ=1.0–1.6 were examined. The second collapse consistently produced the strongest shock activity, and the shock-originating event was observed to lead to microcrack formation. During the second collapse, the shock velocities of 1490–1971 m/s corresponded to peak pressures of 5–464 MPa. To our knowledge, this is the first fluid-dynamic measurement to provide physically reasonable pressure estimates for the second-collapse cavitation shocks at moderate stand-off distances, showing that the damage-producing shocks can reach or exceed the yield strength of technically relevant steels.

## 1 Introduction

Cavitation, the formation and dynamics of vapor bubbles in liquids, underpins a wide range of physical phenomena and technological applications, from propeller erosion to advanced medical therapies. When a cavitation bubble collapses near a solid boundary, the collapse can concentrate energy into a small volume and generate extreme local conditions, including high temperatures, light emission, rapid liquid motion, liquid jets and films, and shock waves [1], [2], [3], [4]. These collapse-induced phenomena are strongly coupled and together determine how mechanical loading is transferred to nearby surfaces. In particular, shock waves are regarded as one of the key mechanisms by which cavitation collapse produces impulsive stresses and initiates material damage [5], [6]. Understanding when and where such shock waves are generated during the collapse process, and how they interact with the wall, is therefore essential for clarifying the physical origin of cavitation-induced surface damage.

Shock waves associated with cavitation have been studied using a variety of optical and acoustic methods. High-speed imaging, including framing and streak photography, has enabled direct visualization of shock-wave emission and propagation in liquids with high temporal resolution [4], [6], [7]. Schlieren and shadowgraph techniques have further revealed shock-wave structures, interactions with bubbles, and reflections from nearby boundaries [8], [9]. Differential interference contrast imaging was used to quantify the wave pressure emitted from a collapsing spherical bubble [10]. More recently, ultra-high-speed cameras [11], femtosecond laser illumination [12], and adaptive optics [13] have improved the ability to resolve rapid density-gradient features in complex cavitation flows. However, much of the existing work has focused on shock waves produced at the initial laser-induced plasma breakdown [14], [15] or on the role of shock waves for bubbles with a very small stand-off distance γ [16], [17], [18] (with γ = D/R_max_, D being the distance of the bubble origin from the wall and R_max_ the maximum radius of the corresponding undisturbed bubble). In contrast, shock-wave generation and evolution during the broader collapse sequence, including their possible contribution to solid-surface damage, remain much less explored.

Interferometric techniques provide a sensitive means of detecting refractive-index (RI) changes associated with density variations and have been applied to time-resolved measurements of shock-wave propagation in transparent media [19], [20], [21], [22]. Nevertheless, such methods require stable optical paths and are highly susceptible to environmental vibrations and phase distortions. These requirements are difficult to satisfy in near-wall cavitation experiments, where strong bubble deformation, rapidly evolving interfaces, scattered light, and mechanical disturbances are intrinsic to the process. Acoustic methods, including needle and fiber-optic hydrophones, have also been used extensively to detect cavitation-induced pressure pulses [23], [24], [25]. However, hydrophones provide point measurements and can disturb the local flow field, while their finite bandwidth and nonlinear response under strong impulsive loading complicate interpretation. As a result, resolving the spatially localized shock-wave activity near a wall during the full collapse process remains challenging.

A major difficulty is that the most relevant region for damage initiation is located very close to the solid–liquid interface, where conventional bulk optical methods often lose sensitivity or suffer from reflections and background contributions. Total internal reflection fluorescence (TIRF) microscopy offers a way to selectively probe this near-wall region. By using an evanescent field for fluorescence excitation, TIRF restricts the observation volume to within a few hundred nanometers of the interface, thereby isolating near-wall flow and shock-wave signatures from the bulk liquid [26]. The basic principle and implementation of TIRF for cavitation studies have been introduced in our previous work [27], where the method was shown to reveal near-wall flow structures and shock-wave features generated by localized collapse events. These observations suggest that TIRF can provide a unique perspective on shock-wave activity directly at the wall, where damage-relevant loading occurs.

In the present study, we use TIRF microscopy combined with synchronized background illumination (BGI) to examine shock waves generated during the collapse and rebound of a near-wall cavitation bubble. We follow shock-wave signatures during successive collapse events and relate their occurrence to the development of surface damage. Shock waves detected within the evanescent field during the first, second, and third collapses are analyzed in terms of their timing, propagation behavior, and radial evolution near the wall. This allows us to take a closer look at the shock-wave phenomena revealed by TIRF and to assess their role in the sequence of events leading to cavitation-induced damage.

## 2 Methods and materials

### 2.1 Optical arrangement

**Fig. 1:**
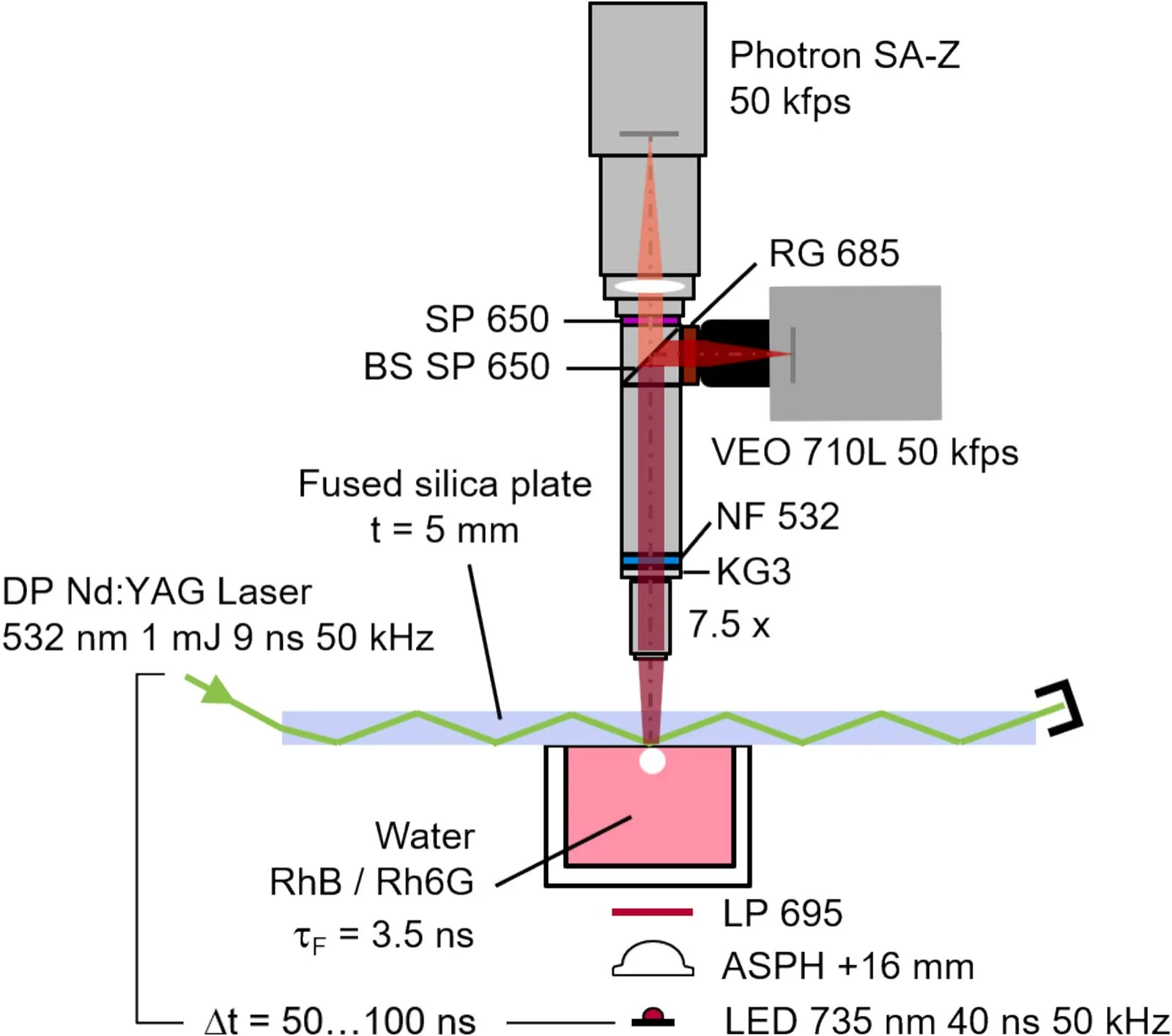
Schematic of the experimental setup. Note that this figure only shows the side view without the bubble-generating optics. The top-view without imaging optics can be found in our previous study [27].

**Fig. 2:**
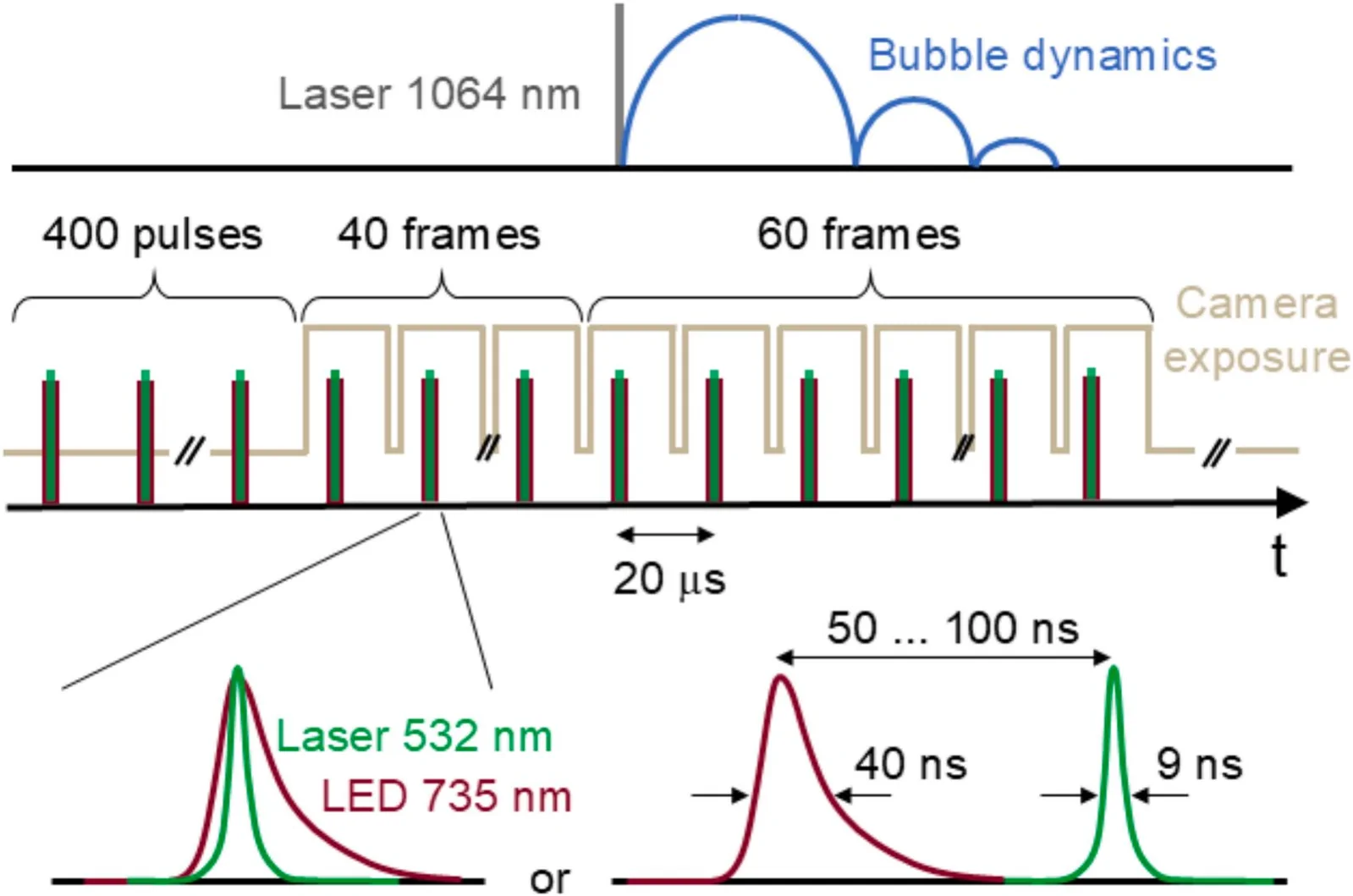
Schematic timing diagram of the experiment.

The experimental setup has been described in detail in our previous work [27]. Here, we provide a brief summary and highlight the newly introduced features. Fig. 1 illustrates the illumination and image detection scheme, while the timing sequence is shown in Fig. 2. The cuvette shown in Fig. 1 was filled with a mixture of 0.4 g/L rhodamine 6G (Rh6G) and 0.4 g/L rhodamine B (RhB) dissolved in distilled water. The rationale behind this particular dye combination in the current work is discussed in Section 2.2. Cavitation bubbles were generated by focusing the output of a Q-switched Nd:YAG laser operating at 1064 nm with a repetition rate of 2 Hz. For TIRF measurements, a pulsed 532 nm laser beam was incident on the front surface of a fused silica plate polished on all sides to achieve total internal reflection at the water–glass interface. The laser pulse repetition rate was 50 kHz. Fluorescence from the dye solution excited by the evanescent wave was imaged on a high-speed camera (Photron SA-Z, 640 × 600 pixels at 50,000 frames/second). Elastic laser light scattering at 532 nm and 1064 nm was suppressed using a notch filter (NF533-17, Thorlabs) and a KG3 filter (Schott), respectively, and a short-pass filter (FESH0650, Thorlabs) blocked LED light (see below). A microscope objective (Mitutoyo M−Plan Apo 7.5 × ) in combination with a tube lens resulted in a projected pixel size of approximately 5 × 5 μm in the object plane.

For the current experiments, the bubble was also background-illuminated (BGI) from below by a red LED (center wavelength 735 nm). The LED was operated with a short-pulse driver (LDP-V 50–100 V3.3, PicoLAS GmbH) with a pulse duration of 40 ns. In the microscope, the transmitted LED light was reflected towards a second high-speed camera (Phantom VEO 710 L). A long-pass filter (RG685, Schott) suppressed fluorescence signal on this camera. At a frame size of 344 × 320 pixels, the camera operated at 50 kfps, corresponding to a projected pixel size of approximately 7.9 × 7.9 μm in the object plane.

A cavitation bubble was generated beneath the polished glass plate every 0.5 s. The bubbles examined in this work had maximum radii of 1–1.5 mm and stand-off distances γ = 1–1.6. The LED and the 532-nm laser were either synchronized to allow simultaneous recording of bubble dynamics or operated with a relative delay of 50–100 ns between BGI and TIRF, as illustrated in Fig. 2. With the exception of the experiments described in Section 2.2, the illumination sources were active for 500 pulses per bubble, corresponding to a duration of 10 ms. 40 frames were recorded before the bubble-generating 1064 nm pulse, while the subsequent 60 frames captured the bubble evolution. In the TIRF images, the pre-bubble frames were used to remove inhomogeneities in the laser intensity profile via flat field correction (FFC), as described in [27]. After FFC, pixel values close to unity represent the undisturbed fluorescent aqueous solution, whereas various events in the bubble create contrast in the form of values above and below unity [27].

### 2.2 Total internal reflection fluorescence

Since the various fluorescence signal dependencies are the subject of the next sections, we briefly review the main equations in the classical (Fresnel-type) description of total internal reflection [27] and those describing fluorescence. TIR occurs when light traveling in a medium 1 with an index of refraction (RI) $n_{1}$ encounters an interface with a medium 2 of lower RI ($n_{1}>n_{2}$) at an angle (normal to the interface) $\theta >\theta_{c}=arcsin\left(n\right)$, where $n=\frac{n_{2}}{n_{1}}$. In our case, at a wavelength of *λ* = 532 nm the RI of fused silica is *n*_1_ = 1.46, the RI of water is *n*_2_ = 1.33, yielding a critical angle of *θ*_c_ = 65.6°.

Near the interface, TIR is accompanied by an evanescent field in medium 2. The EF intensity $I_{\mathrm{EF}}$ decays exponentially with the distance *z* from the interface [28] as given by

(1) $$I_{\mathrm{EF}}=I_{0}e^{-z/d_{p}}$$

where $I_{0}$ is the EF intensity at the wall (z = 0) and *d*_p_ is a characteristic penetration depth. $I_{0}$ is (weakly) polarization-dependent and depends on the incident intensity $I_{\mathrm{in}}$, where ${I_{0}}^{\|}$ is $I_{0}$ in p-polarization, ${I_{0}}^{⊥}$ is in s-polarization.

(2) $${\left(\frac{I_{0}}{I_{\mathrm{in}}}\right)}^{\|}=\frac{4\cos^{2}\theta \left(2\sin^{2}\theta -n^{2}\right)}{n^{4}\cos^{2}\theta +\sin^{2}\theta -n^{2}}$$

(3) $${\left(\frac{I_{0}}{I_{\mathrm{in}}}\right)}^{⊥}=\frac{4\cos^{2}\theta }{1-n^{2}}$$

(4) $$d_{p}=\frac{\lambda_{0}}{4\pi \sqrt{{\left(n_{1}\sin\theta \right)}^{2}-{n_{2}}^{2}}}$$

The EF excites fluorescence in the dye solution, which we collect with the optical efficiency Ω and detect with the photo-electrical efficiency $\eta_{\det}$ with the camera. For weak excitation, the detected fluorescence signal is given by

(5) $$S_{f}=\int_{0}^{\infty }c\left(z\right)I_{\mathrm{EF}}\left(z\right)\sigma_{\mathrm{abs}}{\left(T\right)\Phi }_{f}\left(T\right){\Omega \eta }_{\det}dz$$

where $\sigma_{\mathrm{abs}}\left(T\right)$ is the absorption cross-section of the dye, $\Phi_{f}\left(T\right)$ is the fluorescence quantum yield, c(z) is the fluorophore (dye) volume density, and $I_{\mathrm{EF}}\left(z\right)$ is the EF intensity at distance z from the interface. As indicated in eq. (5), both the absorption cross-section and the fluorescence quantum yield can have a significant dependence on temperature, which will be addressed in Section 2.3. Here, the same fluorophore is used well-mixed throughout the solution, the optical detection path is fixed, and we assume that regardless of where in the EF fluorescence originates it is detected with the same efficiency, such that the corresponding terms are not a function of the wall distance z:

(6) $$S_{f}=\sigma_{\mathrm{abs}}{\left(T\right)\Phi }_{F}\left(T\right)\Omega \eta_{\det}\int_{0}^{\infty }c\left(z\right)I_{\mathrm{EF}}\left(z\right)dz.$$

Considering the line-of-sight integral over z, assuming that the fluorophore volume density is uniform within the EF, i.e., $c\left(z\right)=c_{0}$, and inserting eq. (1) for the EF intensity, we obtain

(7) $$\int_{0}^{\infty }c\left(z\right)I_{\mathrm{EF}}\left(z\right)dz=c_{0}I_{0}\int_{0}^{\infty }e^{-\frac{z}{d_{p}}}dz=c_{0}I_{0}d_{p}.$$

If the relative concentration *R* of dye molecules per water molecule is constant, we can express the dye volume concentration $c_{0}$ in terms of the mass density $\rho_{0}$ of water (molecular weight *M*_w_), which is the quantity that is often used in, e.g., a thermodynamic equation of state

(8) $$c_{0}=\rho_{0}\frac{R}{M_{w}}\propto \rho_{0}.$$

The index “0″ serves as a reminder that these are the values very close to the interface, i.e., in the evanescent field, while the bulk values might be different. The fluorescence signal depends on the fluid density in the evanescent field not only via Eq. (8) but also in that both the EF intensity at the interface $I_{0}$ and the penetration depth $d_{p}$ depend on $n_{2}$ (Eqs. (2), (3), (4)), which in turn can be related to the fluid density via the Lorentz-Lorenz equation, as discussed in Section 3.2. Therefore, the signal is proportional to the product of three factors:

(9) $$S_{f}\left(\rho \right)\propto \;\rho_{0}∙\left[{I_{0}}^{⊥}\left(n_{2}\left(\rho_{0}\right)\right)+{I_{0}}^{\|}\left(n_{2}\left(\rho_{0}\right)\right)\right]∙d_{p}\left(n_{2}\left(\rho_{0}\right)\right)\;.\;\;$$

Each of these factors monotonically increases with density such that overall, the fluorescence signal also monotonically increases (non-linearly) with density. However, the temperature dependence of the fluorescence, as indicated in Eq. (6), potentially complicates the analysis. This is addressed by judicious selection of the dye, as discussed in the next section.

### 2.3 Fluorescence temperature dependence

In our previous study [27], the 50 kHz laser continuously irradiated what was then a Rh6G solution through TIR for several seconds prior to cavitation bubble generation. Temperature-induced variations in fluorescence intensity were observed in the form of radial striations at late stages of bubble collapse in flat-field-corrected fluorescence images. These features were attributed to the convection and mixing of cooler bulk liquid into the laser-heated boundary layer adjacent to the wall. This interpretation was further supported by experiments using RhB solutions, which exhibit an opposite dependence of fluorescence intensity on temperature [27], [29]. Also, the shock waves that will be the central target of this study are expected to increase the local temperature by compression.

To simplify the analysis of the fluorescence signal, the present study uses a temperature-insensitive fluorescent solution. Given the opposing temperature dependencies of Rh6G and RhB fluorescence, the two dye solutions were mixed at different volume ratios. Because the penetration depth of the EF is extremely shallow, conventional temperature measurement techniques, such as thermocouples, are not suitable for this configuration. Therefore, *in situ* measurements were performed to determine the temperature sensitivity of each mixture. The different dye mixtures were placed in the cuvette and continuously TIR-irradiated by the 50 kHz laser beam. The fluorescence excited by each laser pulse was recorded by the high-speed camera over several thousand pulses, presuming that over time the energy input will increase the fluid temperature in a thermal boundary layer that includes the EF.

**Fig. 3:**
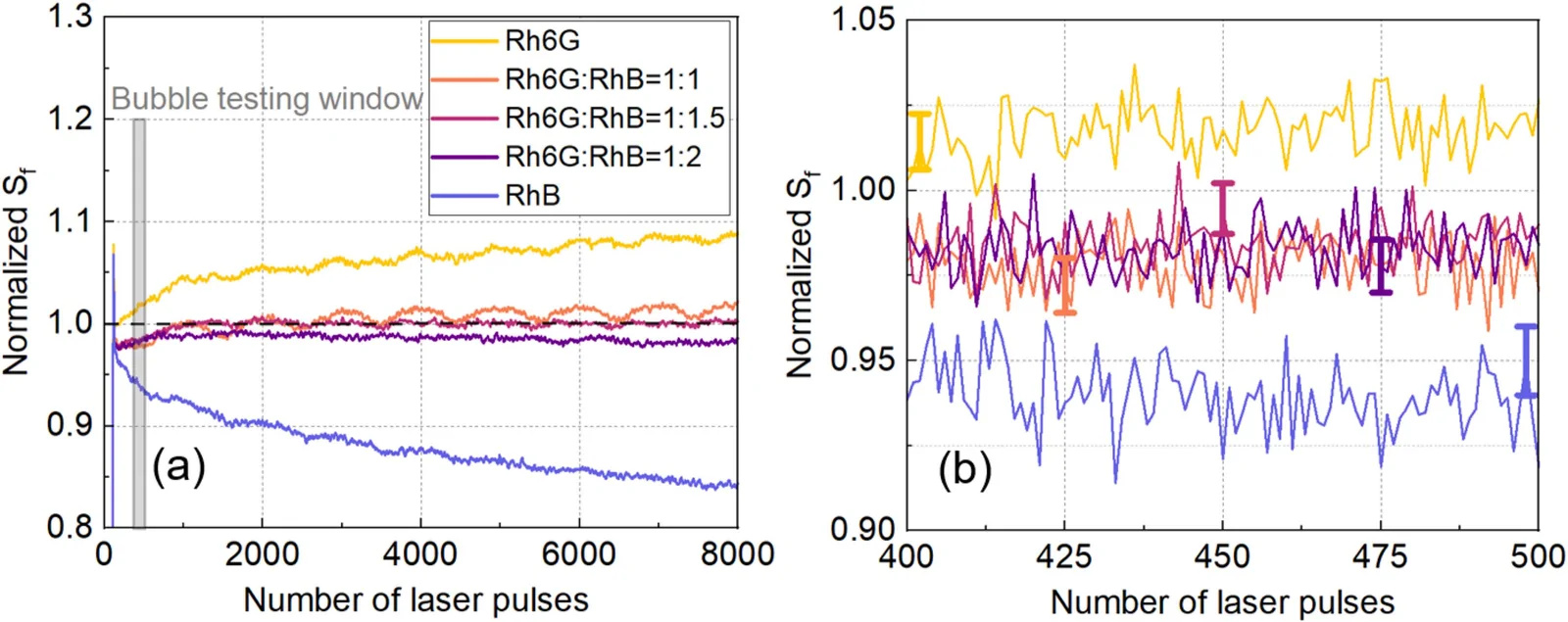
Normalized fluorescence intensity as a function of the number of laser pulses with different volume ratios of 0.4 g/L Rh6G and RhB solutions with (a) a few thousand laser pulses, (b) the 100 pulses indicated by the grey box in (a). The error bars represent the systematic variation among three repetitions.

Fig. 3a presents the fluorescence intensity as a function of laser pulse number for pure 0.4 g/L Rh6G-water and pure 0.4 g/L RhB-water solutions, as well as three mixtures of these two solutions with volume ratios of 1:1, 1:1.5, and 1:2. The periodic fluctuations observed in the traces arise from laser energy fluctuations. The fluorescence intensity was normalized with respect to the average intensity of the first ten laser pulses. For the Rh6G-water solution, the fluorescence intensity increases with pulse number, i.e., increasing temperature, whereas the opposite trend is observed for the RhB-water solution, consistent with previous reports[27], [29]. Based on literature data for Rh6G [29], the fluorescence signal increase from zero to 8000 shots implies a temperature increase of approximately 5 K. For the Rh6G-RhB mixture with a volume ratio of 1:1, the normalized fluorescence signal slightly increases at large pulse numbers compared with the blue dashed line indicating $S_{f}$ = 1, whereas for the 1:2 mixture it shows a slight decrease. Even after more than 1000 laser pulses the signal remains close to unity for the mixture ratio of 1:1.5.

Fig. 3b shows a magnified view of the normalized fluorescence variation within the pulse sequence relevant for the bubble experiments marked in Fig. 3a. The error bars represent the systematic deviations among three independent trials for each mixture. The normalized fluorescence signal slightly but systematically deviates from unity even for the temperature-insensitive mixtures, which is attributed to initially unstable laser energy just after the laser start-up. Nevertheless, each trace remains essentially flat, and the shot-to-shot standard deviation (presumably from random variations in laser energy) over these 100 pulses is about 1 %. Consequently, the Rh6G-RhB mixture with a volume ratio of 1:1.5 was employed in the subsequent experiments to minimize the influence of temperature on the fluorescence signal. We note that the absolute signal was about a factor of 1.8 weaker with this mixture than with Rh6G only, so if the temperature dependence were not an issue, the latter would be the preferred dye.

## 3 Results and discussion

The nomenclature follows our previous study [27]: a “collapse” denotes the contraction of the gas phase to a local spatiotemporal minimum. Unless otherwise specified (e.g., first or second collapse), “collapse” may also refer to the overall collapse process.

### 3.1 Bubble dynamics as seen in simultaneous TIRF and BGI

**Fig. 4:**
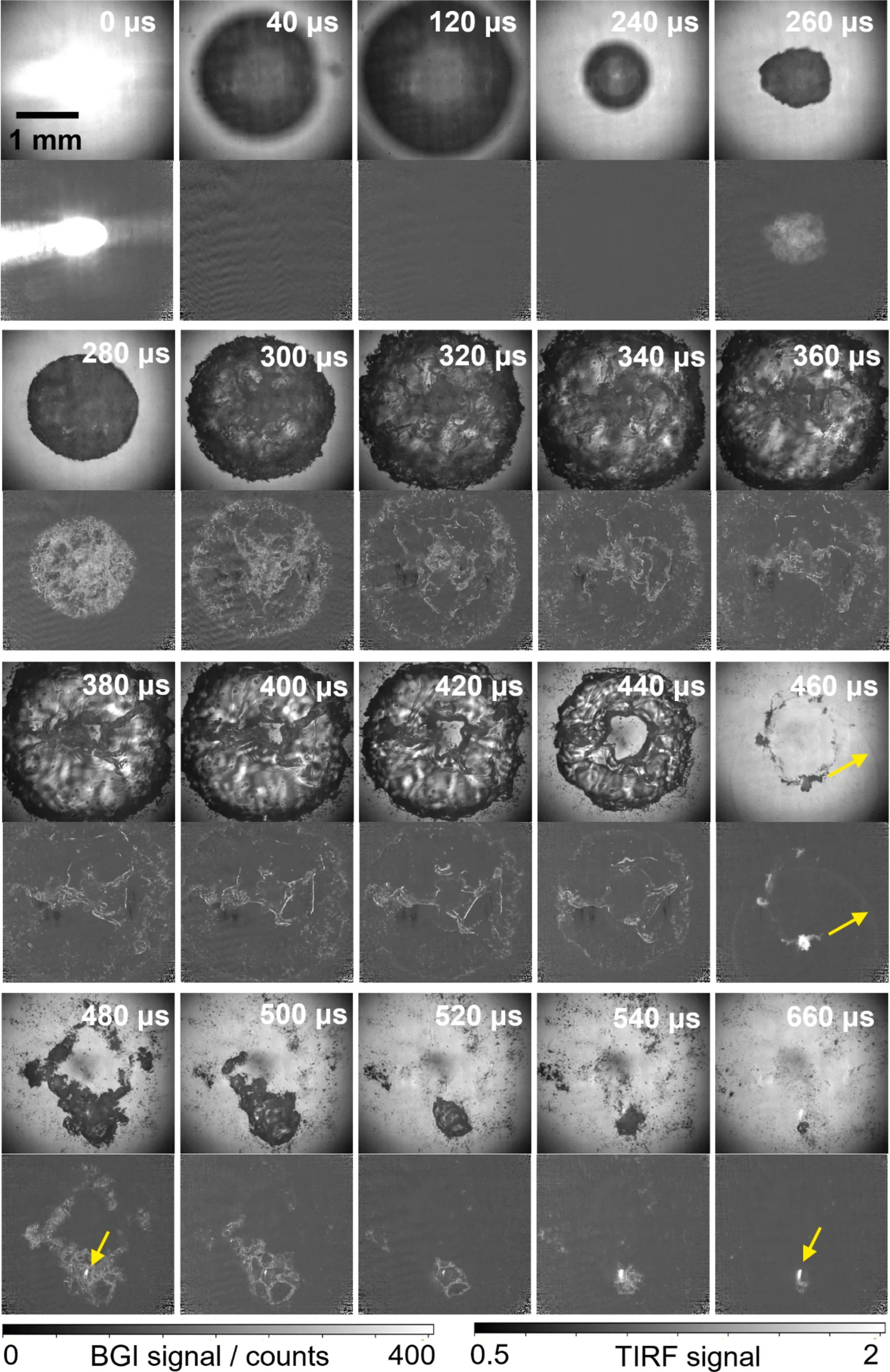
BGI images (top rows) and TIRF images (bottom rows) of a cavitation bubble with r = 1.2 mm, γ = 1.4 under synchronized illumination. Incident angle of the 532 nm laser θ = 70.9 ${\;}^{{}^{\circ}}$. The scale bar indicates the magnification in both image sets. The corresponding video is available in the supplementary materials.

Fig. 4 shows a sequence of selected images giving a top-view overview of the lifecycle of a cavitation bubble with r = 1.2 mm and γ = 1.4. For a sequence with similar parameters, but with the BGI images in the more common side view, we refer to our previous work [27]. Here, in addition to the change in *trans*-illumination orientation, the LED was pulsed much shorter than in that work, resulting in sharp BGI images that can be compared well with their TIRF counterparts. The BGI image sequence by itself is consistent with the literature on single laser-generated bubbles (in pure water) [30], [31]. The laser and LED illumination were peak-synchronized with nanosecond accuracy (see Section 3.3) as shown schematically in the lower-left panel of Fig. 2, which was verified using a photodiode. The laser beam generating the cavitation bubble was incident from the left. In each image pair, the upper row displays the BGI images, while the lower row shows the corresponding (flat-field corrected) TIRF images. The time of the laser-generated plasma is set as 0 µs.

The bubble reaches its maximum radius at 120 µs as indicated by the BGI images. Its edge is out of the shallow depth of focus of the microscope optics. The first collapse occurs between 240 µs and 260 µs as evident from the upper image row. The TIRF image at 260 µs indicates that gas–liquid interfaces of the bubble entered the penetration depth of the evanescent field, which is approximately 115 nm in this configuration according to Eq. (4). For much of the now following re-expansion and re-contraction of the bubble, the TIRF images look quite different from the BGI ones, because the BGI images provide an integrated shadow signal, while the TIRF images only show the bubble interfaces that are close to the wall. From 280 µs to 360 µs, the bubble spreads radially outward, and subsequently transforms into a toroidal shape at 380 µs. The corrugated brighter lines observed between the torus edge and the central jet in the TIRF images from 400 µs to 440 µs are attributed to elongated gas lamellae, as reported in previous studies [27], [32]. This interpretation is supported by the corresponding BGI images.

Following the inward shrinkage of the bubble between 400 µs and 440 µs, the second collapse occurs at 460 μs. It is this moment, the second collapse, that will be examined throughout most of this work. At the 6o’clock position, a nearly perfect circle with an intensity slightly below the background in the BGI image and about 8 % above unity in the TIRF image is observed. This circle is interpreted as a shock wave, with the locally elevated fluorescence signal resulting from shock compression of the dye solution to higher density. TIRF detects the “footprint” of the presumably spherical shock wave on the quartz plate, whereas BGI shows a line-of-sight integrated signal. The shock wave is well-focused in the BGI image, and this is the case for all data we acquired. This implies that the maximum lateral extent of the shock wave is on the quartz plate. Therefore, in the wall-normal direction the origin of the shock wave cannot be very far from the quartz plate. One of the images in our previous study with side-view imaging also showed a second-collapse shock wave that appeared to have originated close to the wall, but a systematic investigation of that feature was not performed in that work[31]. Also consistent with the shock origin being close to the wall, the apparent diameters of the shock waves in the two image modalities (TIRF and BGI) always match. This was used to examine the synchronization, which will be discussed in more detail in Section 3.3. From the current data, we cannot determine whether the shock origin is within the evanescent field.

In the center of the shock wave is a bright spot, representing a maximum of gas concentration along the now fragmented torus where the collapse is locally particularly intense [30], [33]. We also note that in this image pair the greatest similarity in the morphology in the BGI and TIRF images is seen, indicating that all contrast-creating gas–liquid interfaces are relatively close to the wall. The initial appearance of a microcrack is observed at the shock-wave center. This damage is permanent, allowing laser light to “leak” into the fluorescent solution and produce a bright spot due to the violation of the TIR condition. The bright spot is also seen in the BGI images, which is channel cross-talk from TIRF – when the data shown here were acquired, we had not yet added the RG685 filter that provides complete spectral suppression of the shorter-wavelength fluorescence in the BGI images. In this particular sequence in the third collapse at 660 μs, a local concentration of microbubbles again happens to occur close this spot, and the light leakage from the microcrack scatters off it and appears brighter.

In subsequent images, the microcrack persists, and after a few hundred bubbles in visual inspection the quartz plate is seen to be damaged over a more extended area. Inspection of many image sequences reveals that, with these bubbles and imaging parameters, *all* image sequences show shock waves, and always in a single frame during the second collapse. However, not all sequences show a new microcrack, and even fewer have such spatio-temporal coincidence of a shock wave and new microcrack appearing. The first of these qualitative statistical observations is consistent with our findings on ductile materials [31], where the pitting rate, i.e., the average number of new local damage points, could be above or below unity, depending on bubble parameters and material. The second finding we attribute to the fact that at a speed of sound of 1483 m/s it takes less than 1 μs for a shock wave to leave the field of view, while 50 000 frames/second correspond to taking one image every 20 μs. That is, a given image with a newly appeared crack is unlikely to still capture the associated shock wave.

### 3.2 Incident-angle dependence of detecting shock waves by TIRF

**Fig. 5:**
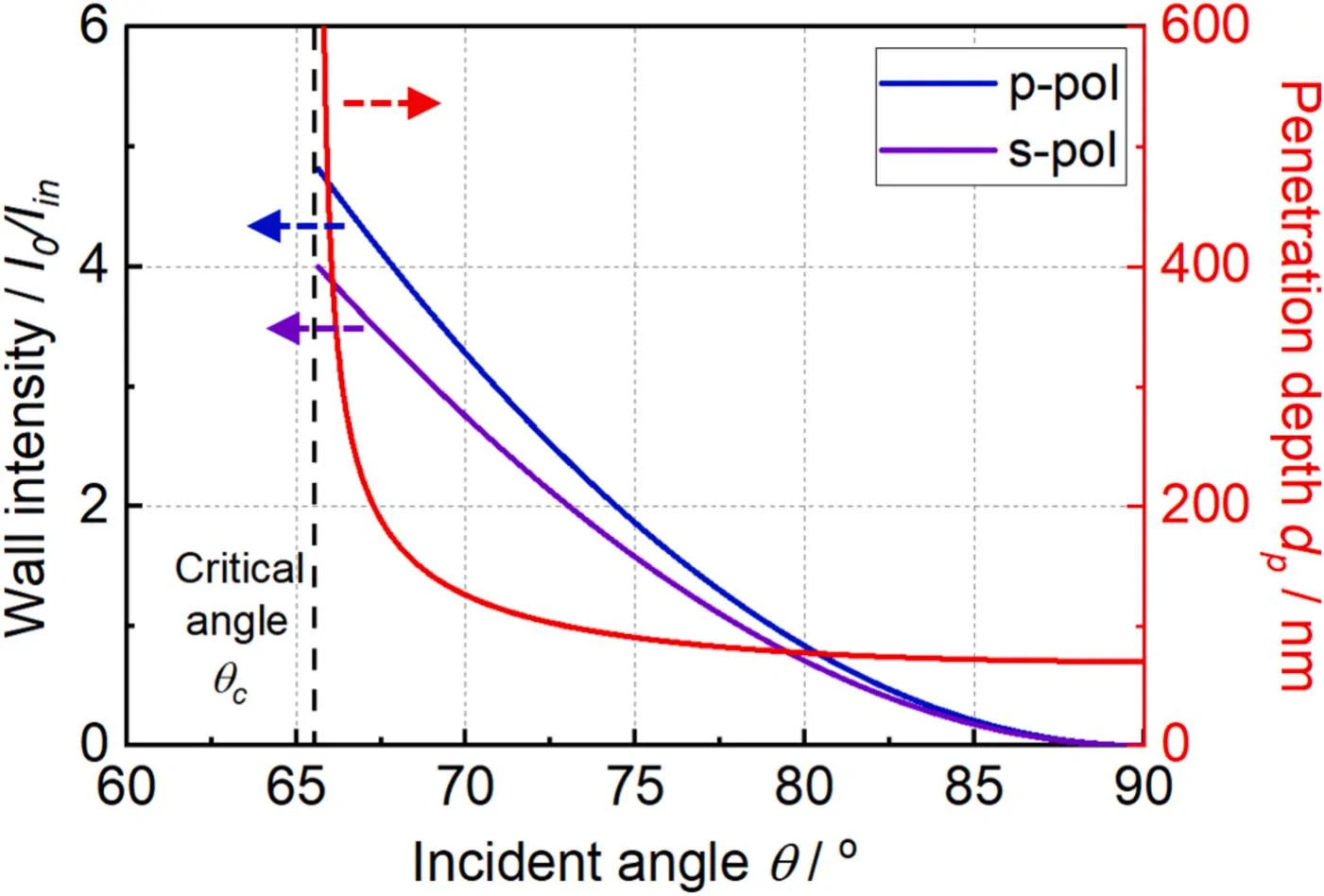
Relative intensity of evanescent field and penetration depth as a function of incident angle. $n_{1}$ = 1.46, $n_{2}$ = 1.33, λ = 532 nm, *θ*_c_ = 65.6°.

It appears that TIRF is very suitable for detecting the footprint of cavitation-induced shock waves. In this section, we explore how the geometry of TIR influences what we observe in the images. Fig. 5 plots the intensity of the EF on the wall $I_{0}$ and the EF penetration depth *d*_p_ as a function of the incident angle θ based on Eqs. (2), (3), (4) with the parameters given in the caption. Both $I_{0}$ and *d*_p_ increase as θ is chosen closer to the critical angle $\theta_{c}$, with *d*_p_ approaching infinity as $\theta \to \theta_{c}$. By adjusting the incident angle, tuning of the EF strength and in particular EF depth can be achieved.

**Fig. 6:**
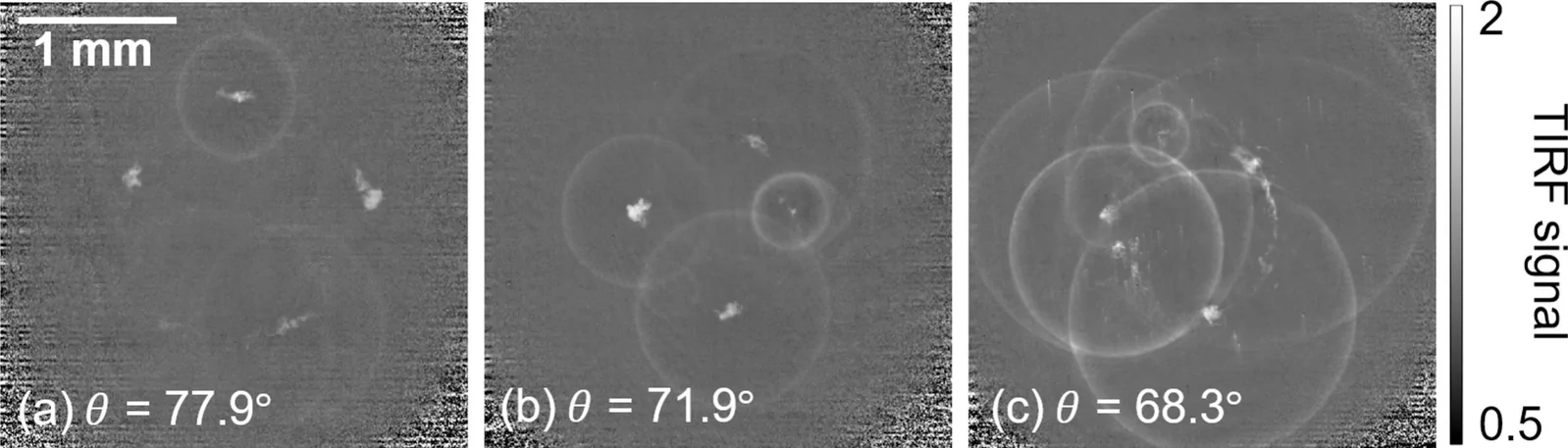
Shock waves in TIRF images at the second collapse of different cavitation bubbles with an incident angle θ of (a) 77.9°, (b) 71.9°, and (c) 68.3°. The corresponding video is available in the supplementary materials.

Fig. 6 shows shock waves during the second collapse at three different incident angles. Each image is from a different bubble, since the laser path had to be adjusted between the experiments, but the images are typical results. Fig. 6 shows that as the incident angle is tuned towards the critical angle, the number of shock-wave rings discernible in an image increases. Since on the scale of the shock-wave radius the EF is very thin regardless of the incident angle, this apparent increase is attributed to the lower detection limit that corresponds to the higher signal and higher signal-to-noise ratio (SNR) as $\theta \to \theta_{c}$. The increase in SNR is clearly visible in the flat-field corrected images from Fig. 6a to Fig. 6c. In our interface-normal detection, the fluorescence signal is depth-integrated such that both the increase in wall intensity and that in EF penetration depth contribute to increasing the signal as $\theta \to \theta_{c}$. Therefore, if stronger fluorescence signal and more sensitive detection are desired, the incident angle should be tuned towards the critical angle. However, the experiment also becomes more sensitive towards alignment errors. Also, very strong shock waves can lead to local image saturation and violation of the TIR condition, as discussed below.

All shock waves in Fig. 6 are centered around bright spots indicating locally intense collapse. The shock-wave radius encodes information about the temporal shock origin while the brightness indicates its relative strength since the fluorescence signal is proportional to local density. The sizes and the center locations of shock circles in Fig. 6 do not show any obvious correlation, implying that at least for these particular bubbles the timing of the asymmetries along the second-collapse torus is stochastic. By eye, there appears to be some correlation between size and brightness of the shock circles, which is expected from the radial spreading and attenuation of the shock waves. In particular Fig. 6c also shows that – as expected – the local density is increased where shock waves overlap, and that the liquid–gas interfaces of the collapse torus disturb the shock-wave propagation.

**Fig. 7:**
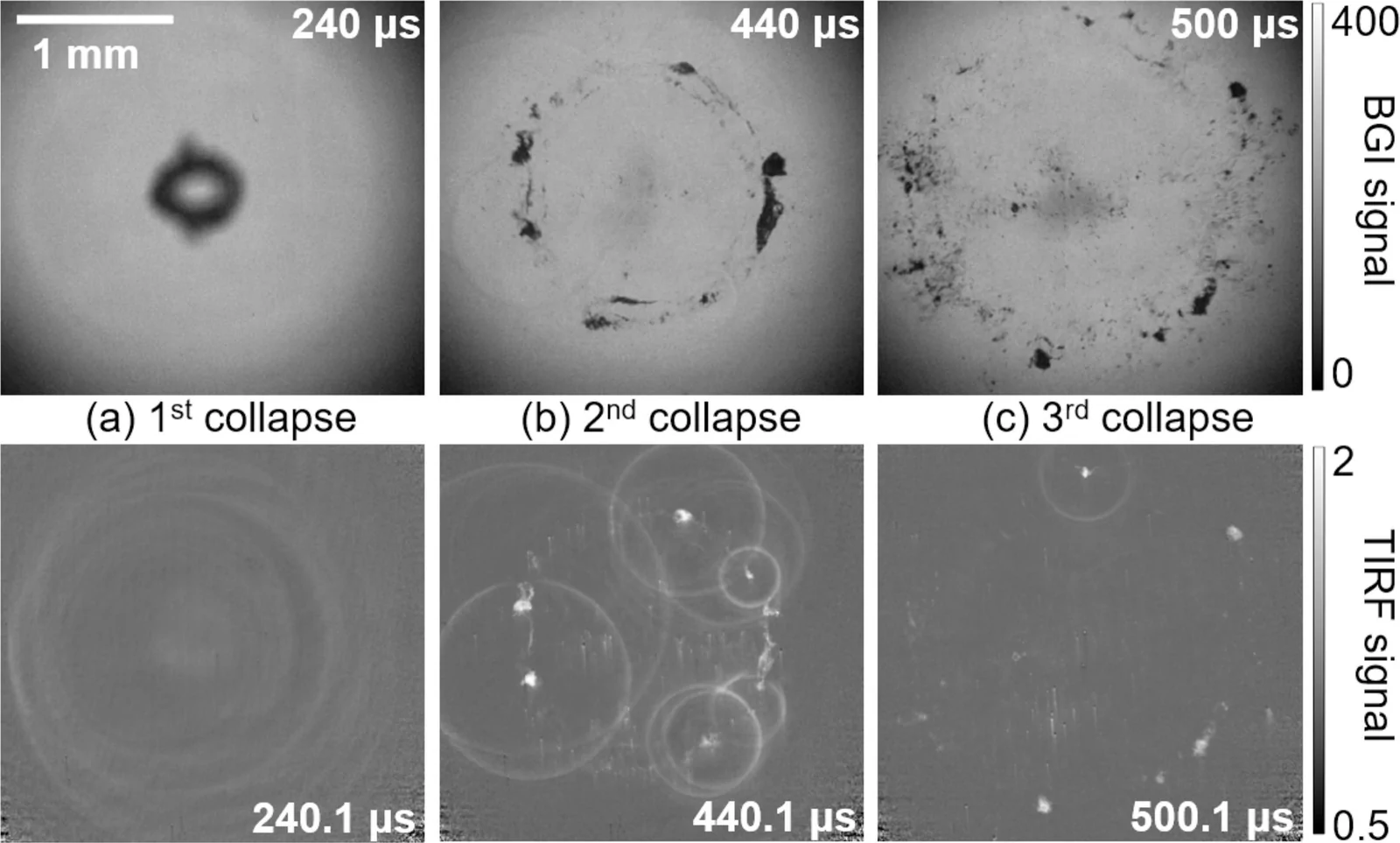
Shock waves in BGI images (top row) and TIRF images (bottom row) of different cavitation bubbles with an incident angle θ of 68.3° at the (a) first collapse, (b) second collapse, and (c) third collapse. γ = 1.4. The BGI images are captured 100 ns prior to the TIRF images. The corresponding video is available in the supplementary materials.

Fig. 7 shows TIRF images of shock waves at different collapse stages recorded with an incident angle of θ = 68.3 ${\;}^{{}^{\circ}}$. As seen in Fig. 7, this angle provides more sensitive detection than the 70.9° used for the image sequence shown in Fig. 4. Here, shock waves generated not only in the second, but also in the first and third collapse, become observable. The stand-off distance of the cavitation bubble in Fig. 7a is γ = 1.4. In this configuration, the first collapse occurs well away from the quartz plate [27], [31] and it cannot be seen in the TIRF images but is identified out of focus in the corresponding BGI sequence. The shock waves appear as several concentric circles with slight spatial offsets, indicating that the first collapse of the cavitation bubble is extended in space and time, the former likely due to asymmetry. Compared to their counterparts in Fig. 7b and c (and Fig. 6), the shock-wave circles in Fig. 7a appear blurred. This is consistent with their origin being away from the quartz plate. That means that the shock wave is not perpendicular to the solid–liquid interface, but at an oblique angle, widening its footprint on the interface. There may also be blurring effects of attenuation and dispersion in the bulk liquid between shock origin and detection plane.

Fig. 7b shows shock waves in the second collapse. The incident angle is the same one as for Fig. 6a, but for this particular bubble, the smaller (younger) shock-wave circles are further to the right than the larger (older) ones, implying a systematic progression of shock-causing local events from left to right. In two cases, at the 2o’clock and the 4o’clock position along the collapse torus, a smaller shock circle is inside a larger one just touching it in one point, which means that it originated when the older shock wave was passing its origin. Overall, for this bubble there appears to be a significant degree of spatio-temporal coherence in the shock origins during the second collapse. While we have not systematically evaluated our current data set for this aspect, the examples in Fig. 6a and 7b seem to be the bracketing cases of second-collapse coherence in our experiment with a stand-off distance of about γ = 1.3. This contrasts with the near complete coherence leading to global shock-focusing observed for γ < 0.3 [16]. It is consistent with the partial shock-wave coherence for γ = 1.08 and 1.45 documented recently via Schlieren imaging by Zhao et al. (see Fig. 12c and d there) [30].

Fig. 7c shows that also the third collapse generates detectable shock waves. As for the second collapse, they appear as sharp-edged rings implying near-wall origin. However, both the number and intensity of shock waves in the third collapse are lower than those observed during the second collapse – the examples shown in Fig. 7c and b, respectively, are typical in this aspect. The reduced shock-wave activity in later collapses is attributed to kinetic energy dissipation during the bubble collapses. Based on both the abundance and strength of shock waves, we conclude that the second collapse is the primary contributor to the damage of the quartz plate used in this study.

**Fig. 8:**
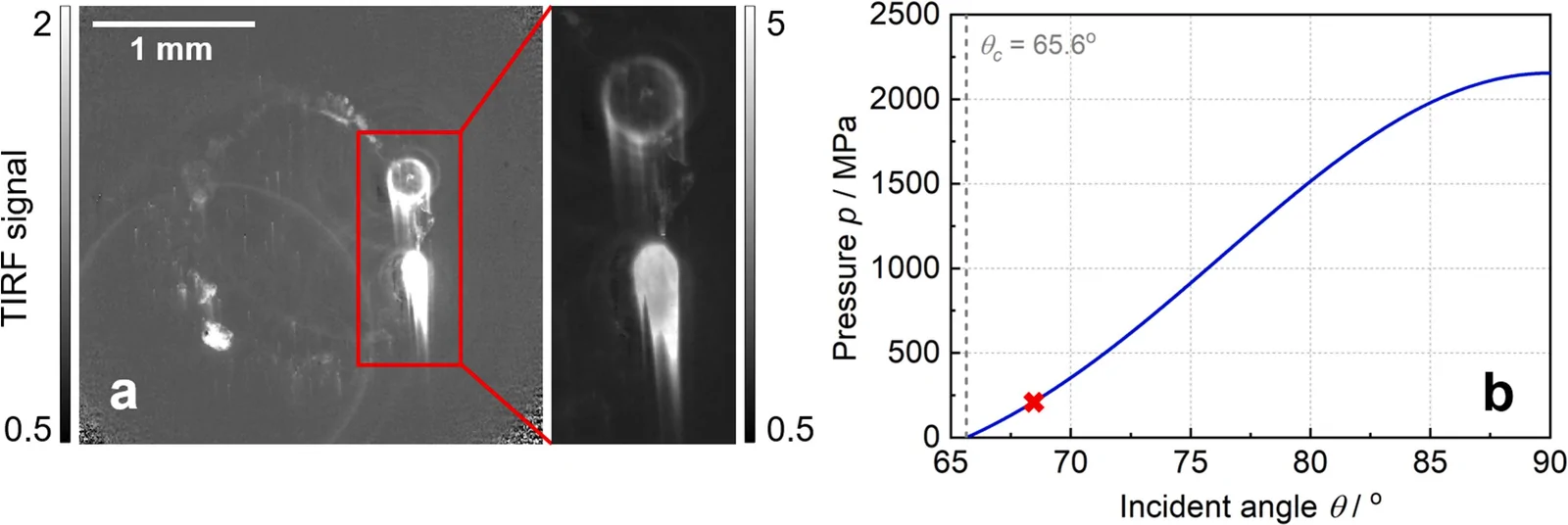
(a) Example TIRF image from the second collapse with two of the shock waves causing light “leakage” into the bulk liquid. (b) Maximum TIRF-detectable shock-wave pressure as a function of incident angle, $n_{1}$ = 1.46, $n_{2}$ = 1.33. The red “x” indicates the incident angle used for the TIRF image in (a).

Fig. 8a displays another interesting phenomenon in the TIRF images. In addition to the large shock-wave circle with relatively weak contrast located at the 7o’clock position of the second-collapse torus, two additional circular features are observed approximately at the 2 and 3o’clock positions. These features have “tails” extending in the direction of the 532 nm laser travel in patterns similar to the microcracks discussed with Fig. 4. The upper feature (at 2o’clock) clearly is a shock wave with a locally intense collapse signature at the center. Light leaks from part of the shock circle. More pronounced leakage is emanating from the lower (3o’clock) feature, which nevertheless has a semicircular shape in the upper portion. The maximum intensity in the upper portion is about 5 times the pre-bubble reference (flat-field) value, and the lower leakage “tail” saturates the sensor. We interpret this as a very young and (still) strong shock. Apparently, for this incident-light angle, the early stage of shock-wave propagation is sufficiently strong to induce a local increase in liquid density and corresponding RI that violates the TIR condition. The differing degrees of leakage observed from the two shock waves are attributed to the sharp pressure decrease that initially occurs over a small radial range.

If light leakage from locally violating the TIR condition is to be avoided, that limits the maximum pressure that can be present on the wall at the time of imaging. At 532 nm, the RI variation of water is primarily governed by density changes. The relation between RI and fluid density ρ is given by the Lorentz-Lorenz equation [34], [35]:

(10) $$A∙\rho =\frac{n^{2}-1}{n^{2}+2},$$

where A is the Lorentz-Lorenz constant. Pressure and density can be related by the Tait equation of state [36]:

(11) $$\frac{p+B}{p_{0}+B}={\left(\frac{\rho }{\rho_{0}}\right)}^{\alpha },$$

where $p_{0}$ and $\rho_{0}$ are the pressure and density at reference conditions, for example, in the EF but ahead of the shock or before the cavitation bubble is created. B is a constant of 314 MPa, and the exponent α is 7 [37], [38].

The index of refraction in the fluid (at reference conditions) is $n_{2}$, and compression by the shock increases that index to $n_{2}+\Delta n_{2}.$ Substitution of Eq. (10) into Eq. (11) then yields:

(12) $$\frac{p+B}{p_{0}+B}={\left(\frac{({n_{2}+\Delta n_{2})}^{2}-1}{{\left(n_{2}+\Delta n_{2}\right)}^{2}+2}∙\frac{{n_{2}}^{2}+2}{{n_{2}}^{2}-1}\right)}^{\alpha }.$$

The limiting case considered here is that the RI of the fluid is increased to the point that the critical angle is equal to the actual incident angle *θ*, i.e., $\Delta n_{2}=\left(n_{1}sin\theta -n_{2}\right),$ where $n_{1}$ = 1.46, $n_{2}$ = 1.33. Thus, the maximum detectable shock pressure is

(13) $$p_{\max}\left(\theta \right)=\left(p_{0}+B\right){\left(\frac{{\left(n_{1}sin\theta \right)}^{2}-1}{{\left(n_{1}sin\theta \right)}^{2}+2}∙\frac{{n_{2}}^{2}+2}{{n_{2}}^{2}-1}\right)}^{\alpha }-B.$$

Fig. 8b plots this maximum pressure for which the shock wave is imaged leakage-free via TIRF versus incident angle. For the incident angle of θ = 68.3${\;}^{{}^{\circ}}$, Fig. 8b indicates that the pressure of both shock waves in Fig. 8a was greater than 200 MPa. In the discussion of Fig. 6 we concluded that incident angles closer to the critical angle are better for investigating weaker shock waves, such as in the later stages of propagation. Additionally, Fig. 8b suggests that larger θ, i.e., farther from the critical angle, is preferable for imaging shock waves in their initial propagation stage.

### 3.3 Pressure estimates based on shock-wave velocimetry

**Fig. 9:**
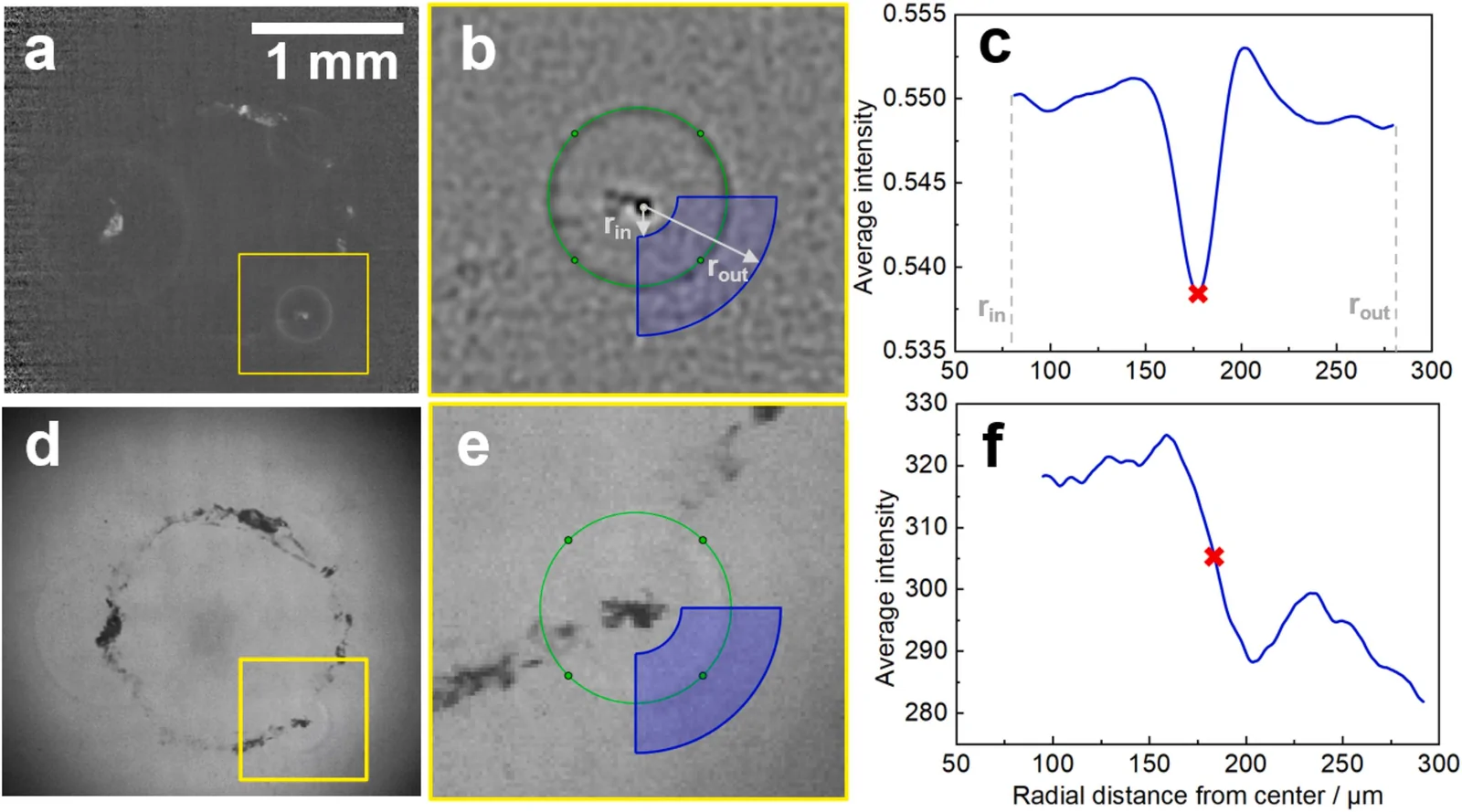
Estimating the shock-wave radius: (a) Example TIRF image and (d) BGI image. (b) and (e) magnifications of the yellow-marked regions in (a) and (d), respectively, with (b) showing the Fourier-based second spatial derivative of (a). The manually labeled shock-wave circle is shown in green and the evaluated arc sectors in blue. (c) and (f) angle-averaged radial profiles from these arc sectors, with the red x indicating the estimated radial shock locations.

The speed of a shock wave is related to its maximum pressure. This relation has been exploited to deduce the shock speed from successive images of shock waves from laser-induced breakdown in water [14], [15]. In this section we establish a workflow to determine the shock-wave radius in both TIRF and BGI images, assess the uncertainty in the synchronization between the two, and then estimate the shock-wave pressure from the velocity measured from time-delayed imaging. Fig. 9 presents a pair of images acquired by TIRF (a) and BGI (d) under synchronized illumination. With respect to shock-wave visualization, BGI is expected to capture the second spatial derivative of the refractive index [39], which is a function of density, whereas the TIRF images directly depend on both RI and density. To establish a consistent baseline between the two techniques, the second-order spatial derivative of the TIRF images under regularized smoothing was computed as shown in Fig. 9a. By visual inspection in a graphic interface, a circle was then positioned on the shock wave as displayed in Fig. 9b. An arc along the circle was selected for analysis. Using the central angle as the independent coordinate, the fluorescence intensity was interpolated at the corresponding pixel positions within the selected sector. The average fluorescence intensity $I_{\mathrm{TIRF}}^{*}$ was obtained by angular averaging. The result is plotted as a function of radial distance from the center in Fig. 9c. The TIRF shock-wave radius $r_{\mathrm{TIRF}}$ was then taken to be the location of the local intensity minimum. An analogous procedure was applied directly to the BGI images (Fig. 9d). The resulting arc-sector averaged radial profile is shown in Fig. 9e. Somewhat unexpectedly, the BGI profile does not resemble the second derivative of the TIRF image, but rather something like a first derivative – indicating that our BGI imaging behaves like Schlieren imaging with respect to the shock-associated RI gradients. Regardless, the midpoint of the region with the steepest intensity gradient was identified as the BGI shock-wave radius $r_{\mathrm{BGI}}$, as shown in Fig. 9f .

**Fig. 10:**
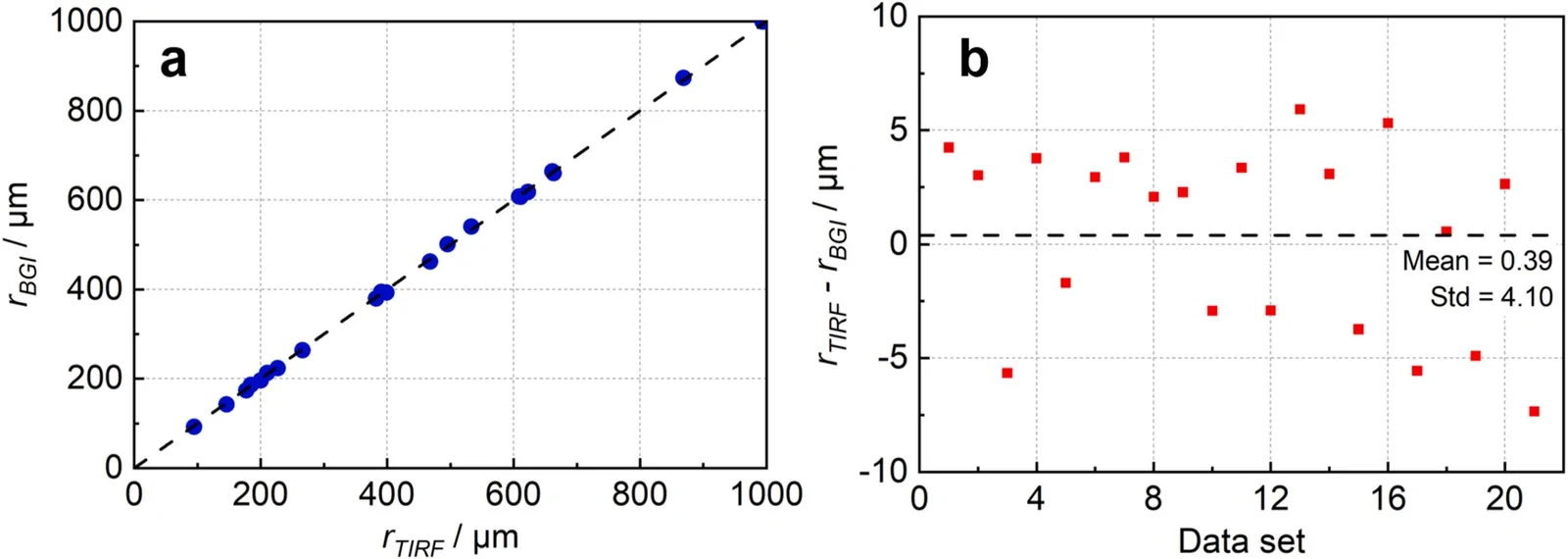
(a) Correlation and (b) difference between measured $r_{\mathrm{TIRF}}$ and $r_{\mathrm{BGI}}$ under synchronized conditions.

The radii of shock waves measured in both TIRF and BGI images were statistically analyzed. Fig. 10a shows the correlation of 21 randomly chosen measured radii $r_{\mathrm{TIRF}}$ and $r_{\mathrm{BGI}}$. All data points are close to the dashed line representing perfect agreement (slope = 1, origin at 0,0). The difference between $r_{\mathrm{TIRF}}$ and $r_{\mathrm{BGI}}$ is shown in Fig. 10b. The offsets are randomly distributed about zero with a mean value of 0.39 μm and a standard deviation of 4.1 μm. The near-zero mean indicates the absence of a systematic bias and suggests that the discrepancy arises primarily from random measurement error. Moreover, the standard deviation is small compared with the shock-wave radii shown in Fig. 10a, confirming that the post-processing method provides enough accuracy for the present analysis.

To determine the shock-wave velocity, a temporal delay Δt ranging from 50 to 100 ns was set between the illumination of the LED and the 50 kHz laser, enabling the acquisition of two temporally correlated shock-wave images. The shock front (wave) velocity u was calculated as:

(14) $$u=\frac{r_{\mathrm{TIRF}}-r_{\mathrm{BGI}}}{\Delta t}.$$

**Fig. 11:**
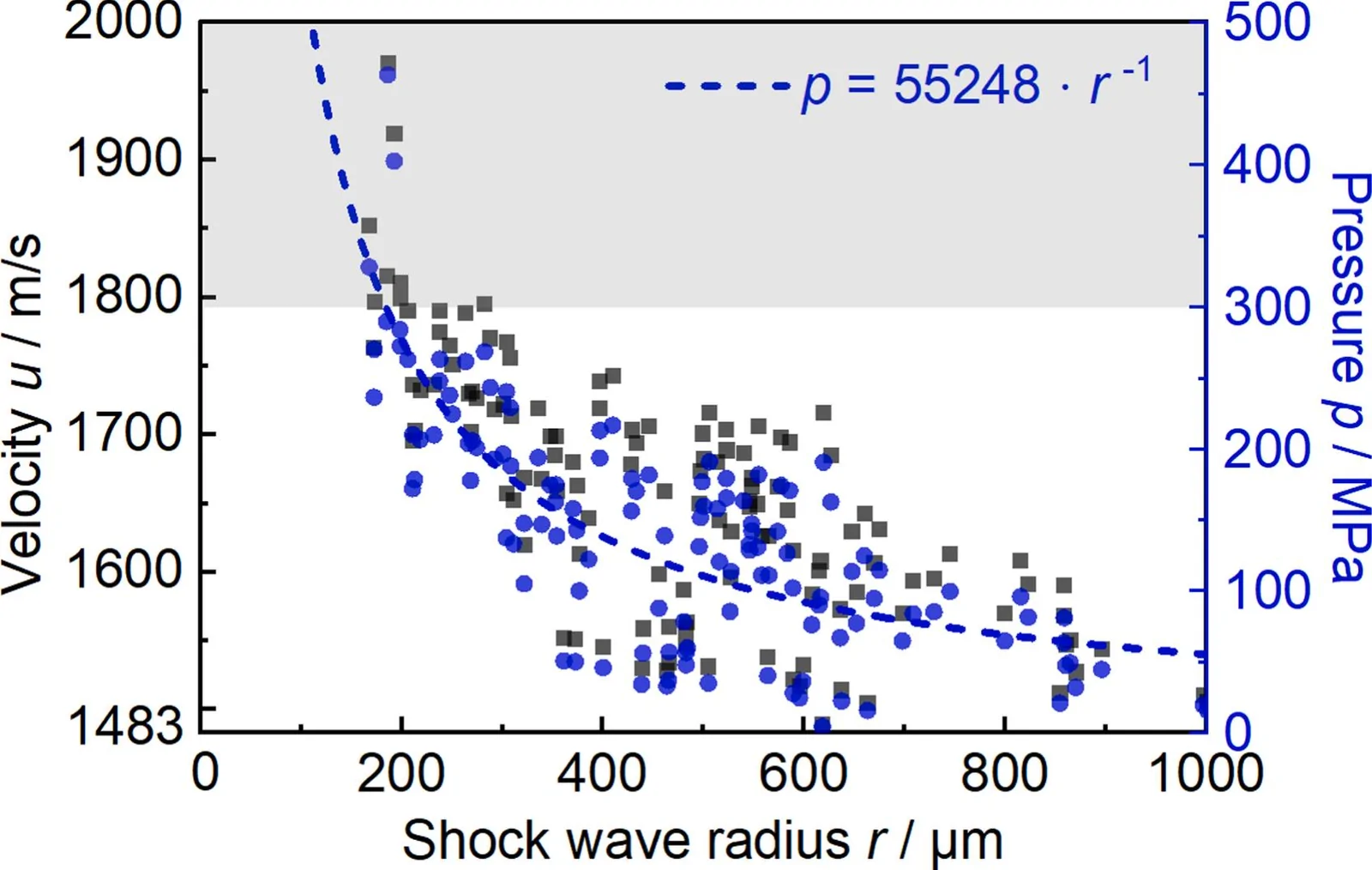
Shock-wave velocity and pressure as functions of radius during the second collapse of the cavitation bubbles with r = 1.0–1.4 mm and γ = 1.2–1.5.

Based on the conservation of momentum at a shock front [40] and the Hugoniot shock relation [41], the relation between u and the shock-wave pressure p is given by [42]:

(15) $$p=c_{1}\rho_{\infty }u\left(10^{\frac{u-c_{\infty }}{c_{2}}}-1\right)+p_{\infty },$$

where the fluid mass density was taken to be the density of water at standard conditions $\rho_{\infty }=$ 998 kg/m^3^, and the speed of sound in water $c_{\infty }$ = 1483 m/s at 20 °C and $p_{\infty }$ = 101 kPa. The empirical constants $c_{1}$ = 5 190 m/s and $c_{2}$ = 25 306 m/s are derived from the Rankine-Hugoniot data [41]. Eq. (15) has been shown to fit experimental data for pressures up to 25 GPa [43]. Fig. 11 presents a scatter plot of shock-wave u and p versus radius *r* in the second collapse, calculated using Eqs. (14), (15). The data points were obtained using a variety of different θ and Δt, rather than from a single experimental run. The shock-wave radius r is set as:

(16) $$r=\frac{r_{\mathrm{TIRF}}+r_{\mathrm{BGI}}}{2}.$$

The measured u ranges from 1490 to 1971 m/s, corresponding to Mach numbers between 1.0 and 1.33, while the associated p spans 5 to 464 MPa. Data points corresponding to shock-wave radii smaller than 150 μm are absent in Fig. 11. This limitation arises primarily from BGI images, which have larger projected pixel size and reduced contrast (due to the LED pulse being longer than the laser pulse) than the TIRF images. Both u and p exhibit a decreasing trend with increasing r. Mur et al. [14] also observed this – physically expected – trend for shock waves produced by laser-induced breakdown. But the data exhibit large scatter in pressure and velocity. For example, at *r* = 400 μ m, *p* = 50… 210 MPa. While there may be systematic errors in the shock-wave relations (Eq. (15)), Fig. 10 suggested low random measurement errors. Therefore, the data scatter reflects the stochastic nature of the shock originating events in the second collapse. This is consistent with the lack of spatio-temporal coherence exemplified by Fig. 6. That figure also showed that varying the incident angle, which determines the weakest TIRF-detectable shock waves, resulted in a pronounced change in the number of detectable shock waves. This sensitivity to the detection limit reflects a broad strength distribution of shock waves generated during the second collapse, again consistent with the stochastic nature of the pressure data in Fig. 11.

Least-squares fitting of the function $p=Ar^{-1}$ to the data yielded A = 55 250 MPa⋅μm (dashed line in Fig. 11). This radius-reciprocal pressure decay is expected for spherical wave propagation when the attenuation of the shock-wave is dominated by geometric spreading, not dissipation.

The highest pressures deduced from the shock speed measurements exceed the yield strength of technical metal alloys. Indicated in Fig. 11 is the range of 300 – 500 MPa that may be considered a reasonable estimate for the yield strength of the stainless steel 316 L as used in our previous work on single-bubble cavitation damage [31]. (The lower end is from a tensile test of that material, the upper from converting the surface hardness measured on the actual samples from that work.) Three data points in Fig. 11 exceed 300 MPa. The corresponding shock waves exhibit strong TIRF signal, consistent with the high pressures inferred from the measured shock velocities. We note that the quasi-steady yield strength may not be a good quantitative metric of material response to the extremely high strain rates expected in a shock impacting the solid surface. However, the data imply that for radii comparable to the few micrometers lateral extent of a pit on a technical alloy [5], [31], shock pressures could be much higher. It is not clear down to how small radii the shock-wave attenuation is dominated by geometric spreading, but assuming the fit in Fig. 11 holds to 50 μm radius – much larger than any single pit seen on 316 L for the relevant bubble parameters –, that radius corresponds to a pressure of 1.1 GPa.

Our shock-speed deduced pressures are more than an order of magnitude higher than literature values measured with pressure sensors during the second collapse of bubbles with similar stand-off distances, by Tomita and Shima in 1986 and very recently by Subramanian et al. in 2026, 11 – 12 MPa for γ = 1.1 – 1.2 and r_max_ = 5 – 6 mm[44], [45]. We attribute that to the large size of pressure sensors used in these studies (2.5 mm[45] and 5.55 mm[44] diameter) that leads to spatial averaging of the very local peak pressure in the shock. The current shock pressure estimates align much better with the results that Pöhl et al.[46] obtained from an inverse finite-element (FEM) analysis of pits on pure copper after short ultrasonic cavitation. They estimate that peak pressures of 2.4 – 3.5 GPa caused the typically 6 μm wide and 150 nm deep pits they experimentally found. However, the best fit of FEM with the experimental pit geometry was achieved with load profiles with a width similar to the pit itself, and it is not possible to extrapolate to such small radii from the current data with any certainty.

Since the shock-originating events are due to the at least somewhat stochastic circumferential break-up of the second-collapse torus, it is not to be expected that axisymmetric numerical simulations can reproduce these features. There are a few three-dimensional simulations of single-bubble collapse[30], [47], [48]. Recently, Zhao et al.[30] simulated bubbles with γ = 0.72 and r_max_ = 0.53 mm, including bubbles with various degrees of slight initial asymmetry (as in real laser-induced bubbles). They find the highest pressures in the second collapse 75 μm away from the wall at 192 MPa, and – starting from a somewhat different initial asymmetry – a maximum wall pressure of 94 MPa. These pressures are on the same order as our measurements, but still too low to deform technical alloys. This may be because their bubbles were smaller, and smaller bubbles are known to produce less damage[31], or because of numerical dissipation affecting the simulated shock waves.

Finally, we note that the local peak temperature in the shock is not particularly high. Assuming adiabatic – but not necessarily isentropic – shock heating from 1 bar and 20 °C, the shock-wave data of Rice et al.[41] yield a temperature increase of 16 K for a shock pressure of 500 MPa, slightly above the highest pressures we deduced from the shock velocities (see Fig. 11). Again, extrapolation to much smaller radii is highly uncertain, but at least this estimate implies that the fluorescence behavior of the dye mixture will not show any gross deviation from 5 K heating in the multi-shot heating experiment discussed in Section 2.3.

## 4 Conclusions

This study develops total internal reflection fluorescence (TIRF) into a diagnostic tool for visualizing shock waves created by collapsing laser-induced cavitation bubbles in rhodamine-dyed water. We combined this imaging mode with short-pulsed transillumination on a second camera to show a more traditional view of the bubble dynamics in the same plane. The fluorescence imaging senses the compression of the fluid by spherically expanding shock waves close to the interface of a flat solid–liquid boundary (a fused silica plate) near the collapsing bubble. Across the propagating shock, temperature, pressure, and mass density, and thereby optical density, change, which can be sensed by a change in local fluorescence signal intensity.

To simplify the dependency of the fluorescence signal on the thermofluidic state, the influence of temperature was eliminated by choosing a mixture of Rhodamine 6G and Rhodamine B such that their opposing trends in quantum efficiency with temperature cancel. Mass density influences the fluorescence signal both via the dye volume concentration and the index of refraction. We show how via tuning the laser incidence angle with respect to the critical angle, control of the evanescent field depth and density sensitivity in the TIRF images is achieved. This allows robust detection of the “footprint” of shock waves from different collapse stages. Strong shocks at small radii locally increase the refractive index enough to violate the TIR condition, producing characteristic laser leakage streaks in the fluorescence images.

Bubbles with a radius of 1–1.5 mm and moderate non-dimensional wall stand-off distance γ = 1.0–1.6 were examined. While shock waves from the first and third collapse were observed, their footprint on the wall is weak because they originate farther from the glass wall. In contrast, the second collapse consistently produced the strongest and most abundant shock waves. We see instances of microcracks appearing in the glass plate in exact spatio-temporal coincidence with the shock-wave origin during the second collapse, which strongly suggests that the shock waves are the cause of the glass damage. For the chosen stand-off distances, the shock patterns suggest a partially coherent collapse of the then toroidal bubble, consistent with recent detailed experimental–numerical investigations [30]. The pronounced self-focusing documented in the literature[16], [18] for much smaller stand-off distances occurred only occasionally over limited sectors of the torus.

The peak pressure in the shock was estimated from the shock-wave speed. To this end, the TIRF laser illumination was time-delayed with respect to the LED *trans*-illumination to yield a series of image pairs from which the difference in shock radii and thus the radial propagation velocity was estimated. In the second collapse, velocities of 1490 – 1971 m/s (Ma = 1 – 1.33) translate via a semi-empirical state equation to pressures of 5–464 MPa, with pressure scaling as $p\propto r^{-1}$. This is consistent with spherical geometric spreading and rapid attenuation toward linear acoustic behavior. For the smallest radii that could be accurately measured, the pressure is thus on the order of the yield strength of technically relevant steels. To the best of our knowledge, this is the first direct, i.e., fluid-dynamic, measurement of such high pressures in the second collapse, which for bubbles with moderate stand-off distances is the damage-relevant part of the collapse process. Since the shock waves for which we were able to measure the shock speed and thus pressure had already expanded to at least 150 μm radius, the corresponding pressures are only a *lower* bound of the actual peak pressures where the solid is nearest to the shock-originating event. This is consistent with the observed breakdown of the TIR condition, indicating locally high fluid density, for the smallest-radii shocks observed. Taken together, the evidence in this work thus strongly suggests that at least one of the mechanisms of initial damage creation in the second collapse for moderate stand-off distances is the locally strong collapse of torus fragments radiating out shock waves that carry enough pressure to microscopically crack or (in the case of ductile materials) plastically indent the nearby solid surface.

## CRediT authorship contribution statement

**Fangyi Wang:** Writing – original draft, Software, Methodology, Investigation, Formal analysis, Data curation. **Sebastian A. Kaiser:** Writing – review & editing, Validation, Supervision, Methodology, Funding acquisition, Conceptualization.

## Funding

This work was funded by the 10.13039/501100001659German Research Foundation, DFG project ID 451715773.

## Declaration of competing interest

The authors declare that they have no known competing financial interests or personal relationships that could have appeared to influence the work reported in this paper.

## References

[1] Suslick K.S., Flannigan D.J. (2008). Inside a collapsing bubble: sonoluminescence and the conditions during cavitation. Annu. Rev. Phys. Chem..

[2] Blake J.R., Gibson D. (1987). Cavitation bubbles near boundaries. Annu. Rev. Fluid Mech..

[3] Ohl C.-D., Kurz T., Geisler R., Lindau O., Lauterborn W. (1999). Bubble dynamics, shock waves and sonoluminescence. Philos. Trans. Math. Phys. Eng. Sci..

[4] Vogel A., Lauterborn W., Timm R. (1989). Optical and acoustic investigations of the dynamics of laser-produced cavitation bubbles near a solid boundary. J. Fluid Mech..

[5] Philipp A., Lauterborn W. (1998). Cavitation erosion by single laser-produced bubbles. J. Fluid Mech..

[6] Supponen O., Obreschkow D., Kobel P., Tinguely M., Dorsaz N., Farhat M. (2017). Shock waves from nonspherical cavitation bubbles. Phys. Rev. Fluids.

[7] Bokman G.T., Biasiori-Poulanges L., Lukić B., Bourquard C., Meyer D.W., Rack A., Supponen O. (2023). High-speed x-ray phase-contrast imaging of single cavitation bubbles near a solid boundary. Phys. Fluids.

[8] Yamamoto S., Tagawa Y., Kameda M. (2015). Application of background-oriented schlieren (BOS) technique to a laser-induced underwater shock wave. Exp. Fluids.

[9] Lindau O., Lauterborn W. (2003). Cinematographic observation of the collapse and rebound of a laser-produced cavitation bubble near a wall. J. Fluid Mech..

[10] Kordel S., Hussong J. (2018). Utilizing differential interferometry for spatially resolved pressure field measurements of laser-induced cavitation. Exp. Fluids.

[11] Mur J., Agrež V., Ohl C.-D., Petkovšek R. (2026). Cavitation erosion from single acoustically driven bubbles. Ultrason. Sonochem..

[12] Reuter F., Ohl C.-D. (2021). Supersonic needle-jet generation with single cavitation bubbles. Appl. Phys. Lett..

[13] Agrež V., Požar T., Petkovšek R. (2020). High-speed photography of shock waves with an adaptive illumination. Opt. Lett..

[14] Mur J., Reuter F., Kočica J.J., Lokar Ž., Petelin J., Agrež V., Ohl C.D., Petkovšek R. (2022). Multi-frame multi-exposure shock wave imaging and pressure measurements. Opt. Express.

[15] Reuter F., Mur J., Petelin J., Petkovsek R., Ohl C.-D. (2024). Shockwave velocimetry using wave-based image processing to measure anisotropic shock emission. Phys. Fluids.

[16] Reuter F., Deiter C., Ohl C.-D. (2022). Cavitation erosion by shockwave self-focusing of a single bubble. Ultrason. Sonochem..

[17] Yang C.X., Ma J.M., Wu J., Wen H.G., Xiao R.F., Wang F.J., Yao Z.F. (2025). Experimental Investigation into Cavitation erosion Characteristics of Stainless Steel Caused by Laser-Induced Bubbles. J. Fluids Eng..

[18] Mur J., Petkovsek R., Ohl C.-D. (2026). Sonoluminescence from single cavitation bubbles near solid surfaces. Ultrason. Sonochem..

[19] Ward B., Emmony D.C. (1991). Direct observation of the pressure developed in a liquid during cavitation‐bubble collapse. Appl. Phys. Lett..

[20] Mauger C., Méès L., Michard M., Azouzi A., Valette S. (2012). Shadowgraph, Schlieren and interferometry in a 2D cavitating channel flow. Exp. Fluids.

[21] Kim T., Liang J., Zhu L., Wang L.V. (2020). Picosecond-resolution phase-sensitive imaging of transparent objects in a single shot. Sci. Adv..

[22] Wilson B., Fan Z., Sreedasyam R., Botvinick E., Venugopalan V. (2021). Single-shot interferometric measurement of cavitation bubble dynamics. Opt. Lett..

[23] Arvengas A., Davitt K., Caupin F. (2011). Fiber optic probe hydrophone for the study of acoustic cavitation in water. Rev. Sci. Instrum..

[24] Lokar Ž., Petelin J., Horvat D., Agrež V., Petkovšek R. (2026). Laser induced cavitation bubble and shockwave measurements with fiber optical hydrophone very close to origin. Exp. Therm Fluid Sci..

[25] Osterman A., Hočevar M., Širok B., Dular M. (2009). Characterization of incipient cavitation in axial valve by hydrophone and visualization. Exp. Therm Fluid Sci..

[26] Kazoe Y., Yoda M. (2013). Evanescent Wave-based Flow Diagnostics. J. Fluids Eng..

[27] Wang F., Kühlmann J., Kaiser S.A. (2025). Near-wall dynamics of single cavitation bubbles imaged by total internal reflection fluorescence. Ultrason. Sonochem..

[28] Axelrod D. (1981). Cell-substrate contacts illuminated by total internal reflection fluorescence. J. Cell Biol..

[29] Kuzkova N., Popenko O., Yakunov A. (2014). Application of temperature-dependent fluorescent dyes to the measurement of millimeter wave absorption in water applied to biomedical experiments. Int. J. Biomed. Imaging.

[30] Zhao Z., Li S., Xiong C., Cui P., Wang S., Zhang A.M. (2025). New insights into the cavitation erosion by bubble collapse at moderate stand-off distances. J. Fluid Mech..

[31] Kühlmann J., Kaiser S. (2024). Single-bubble cavitation-induced pitting on technical alloys. Tribol. Lett..

[32] Reuter F., Kaiser S.A. (2019). High-speed film-thickness measurements between a collapsing cavitation bubble and a solid surface with total internal reflection shadowmetry. Phys. Fluids.

[33] Kühlmann J., Lozano C., Hanke S., Kaiser S. (2023). Correlation of laser-induced single bubbles with cavitation damage via in-situ imaging. Wear.

[34] Lorentz H. (1880). Uber die Beziehung zwischen der Fortpflanzung des Lichtes und der Körperdichte (about the relationship between the propagation of light and the body density). Wiedemann Ann.

[35] Lorenz L. (1880). Ueber die Refractionsconstante. Ann. Phys..

[36] P.G. Tait, Report on some of the physical properties of fresh water and of sea water: Johnson Reprint Corporation, 1888.

[37] Hayasaka K., Tagawa Y., Liu T., Kameda M. (2016). Optical-flow-based background-oriented schlieren technique for measuring a laser-induced underwater shock wave. Exp. Fluids.

[38] Brujan E. (2010).

[39] G.S. Settles, Basic Concepts. Schlieren and Shadowgraph Techniques: Visualizing Phenomena in Transparent Media. Edited by Settles GS. Berlin, Heidelberg: Springer Berlin Heidelberg, 2001. pp. 25–38.

[40] G. Duvall, G. Fowles, Shock waves. High Pressure Physics and Chemistry, Volume 2 1963, 2:209.

[41] Rice M.H., Walsh J.M. (1957). Equation of state of water to 250 kilobars. J. Chem. Phys..

[42] Vogel A., Busch S., Parlitz U. (1996). Shock wave emission and cavitation bubble generation by picosecond and nanosecond optical breakdown in water. J. Acoust. Soc. Am..

[43] Brujan E.A., Ikeda T., Matsumoto Y. (2008). On the pressure of cavitation bubbles. Exp. Therm Fluid Sci..

[44] Tomita B.Y., Shima A. (1986). Mechanisms of impulsive pressure generation and damage pit formation by bubble collapse. J. Fluid Mech..

[45] Subramanian R.K., Yang Z., Romanò F., Coutier-Delgosha O. (2026). Single bubble collapse near a wall: pressure wave and microjet impact. Phys. Fluids.

[46] Pöhl F., Mottyll S., Skoda R., Huth S. (2015). Evaluation of cavitation-induced pressure loads applied to material surfaces by finite-element-assisted pit analysis and numerical investigation of the elasto-plastic deformation of metallic materials. Wear.

[47] Sagar H.J., Hanke S., Underberg M., Feng C., el Moctar O., Kaiser S.A. (2018). Experimental and numerical investigation of damage on an aluminum surface by single-bubble cavitation. Mater. Perform. Charact..

[48] Sagar H.J., el Moctar O. (2020). Dynamics of a cavitation bubble near a solid surface and the induced damage. J. Fluids Struct..

